# Effect of Storage Duration on Amylase, Protease, and Lipase Activities in Ultrasound-Assisted Extracted Bovine Pancreatin

**DOI:** 10.3390/molecules31060980

**Published:** 2026-03-15

**Authors:** Gulmira Kenenbay, Urishbay Chomanov, Gulzhan Zhumaliyeva, Alibek Alashevich Tursunov

**Affiliations:** Branch of the “Kazakh Research Institute of Processing and Food Industry”, Almaty 050000, Kazakhstan; gkenenbay@mail.ru (G.K.); chomanov_u@mail.ru (U.C.); guljan_7171@mail.ru (G.Z.)

**Keywords:** storage duration, microbiological parameters, bovine pancreatin, protease, amylase, lipase, enzymatic activity

## Abstract

Long-term stability of multienzyme protein systems is governed by preservation of conformational integrity and resistance to thermally induced structural destabilization. This study evaluated bovine pancreatin (BP) obtained by conventional extraction (CM) and ultrasound-assisted extraction (UAM) during 0–930 days of storage at 10–40 °C. Amylolytic (AA), proteolytic (PA), and lipolytic activities (LA), representing the functional enzymatic activity (EA) of the multienzyme protein system, were monitored to characterize degradation kinetics and activity loss associated with conformational destabilization. After 930 days at 20 ± 1 °C, UAM retained 76% of initial AA compared with 58% for CM, corresponding to a 31% higher residual activity in UAM. LA demonstrated comparatively high stability in both preparations (~84% retention), whereas PA exhibited delayed degradation and significantly higher residual values in UAM samples. Two-way ANOVA confirmed significant effects of extraction method, storage duration, and their interaction (*p* < 0.001), indicating method-dependent kinetic behavior. Elevated temperatures (35–40 °C) accelerated inactivation, consistent with increased molecular mobility and reduced conformational stability. The smoother degradation trajectories and lower apparent inactivation rates observed in UAM preparations suggest kinetic stabilization, potentially associated with improved conformational preservation and reduced extraction-induced structural stress. Both preparations complied with pharmacopoeial microbiological limits. These findings support the hypothesis that UAM enhances long-term functional stability of complex multienzyme systems through mechanisms related to conformational resilience.

## 1. Introduction

Pancreatin is a complex multienzyme preparation of animal origin widely used in food processing, pharmaceutical formulations, and enzyme-assisted industrial technologies. As a system composed primarily of α-amylase, serine proteases (trypsin, chymotrypsin, elastase), and lipases, its functional performance depends on preservation of structural integrity and catalytic activity during storage [1,2]. Owing to its proteinaceous nature, pancreatin is inherently sensitive to environmental factors such as temperature, moisture, water activity, and oxygen exposure, which may induce conformational destabilization, aggregation, autolytic degradation, and progressive enzymatic inactivation [3,4,5].

From a thermodynamic perspective, long-term protein stability is governed by the Gibbs free energy difference between native and partially unfolded conformational states. Elevated temperature and increased molecular mobility may reduce the energetic barrier separating these states, thereby increasing the population of conformationally labile intermediates. Protein stability can be interpreted within an energy landscape framework, where storage-induced perturbations shift the equilibrium toward partially unfolded conformational ensembles with reduced catalytic competence. In hydrated systems, water-mediated conformational mobility and disruption of intramolecular hydrogen-bond networks may further facilitate structural rearrangements and aggregation pathways [4,5].

In addition to physicochemical stability, microbiological quality is critical for maintaining product safety and functional performance [6,7]. Elevated moisture and water activity promote microbial proliferation and may accelerate structural degradation of enzymes, thereby compromising both stability and regulatory compliance. Consequently, long-term preservation of EA—particularly that of α-amylase, proteases, and lipases—represents a key indicator of pancreatin quality during storage [8,9,10].

At the molecular level, stability of multienzyme preparations depends on maintenance of tertiary structure, metal-ion coordination, and resistance to proteolytic self-digestion. PA in pancreatin is mainly attributed to serine endopeptidases, whereas α-amylase is a calcium-dependent metalloenzyme requiring structural Ca^2+^ ions for conformational stability and catalytic competence [11,12]. Disruption of tertiary interactions or metal-ion binding may reduce thermostability and accelerate activity decline. Because pancreatin represents a heterogeneous multienzyme system, its overall stability reflects the combined behavior of structurally distinct catalytic proteins rather than a single enzymatic component [13,14,15].

Although previous investigations have addressed short-term stability, thermal resistance, and pH-dependent activity of individual pancreatic enzymes, systematic long-term studies integrating storage duration, temperature effects, extraction technology, and microbiological quality assessment remain limited. In particular, quantitative data describing degradation kinetics in complex multienzyme systems over extended storage periods are scarce [2,3]. This lack of integrated long-term evaluation represents a significant gap for pharmaceutical and biotechnological applications requiring predictable shelf-life and regulatory compliance [16,17].

UAM has emerged as a technique to enhance enzyme recovery from biological tissues. Its mechanism involves acoustic cavitation, where formation and collapse of microbubbles generate localized shear forces and transient microenvironments that improve mass transfer and facilitate enzyme release [17,18]. While UAM has been extensively investigated in terms of extraction efficiency and immediate catalytic enhancement, its potential influence on long-term stability of multienzyme preparations has not been systematically evaluated. For temperature- and moisture-sensitive systems such as pancreatin, understanding whether extraction technology affects subsequent storage behavior is of practical and scientific relevance [19,20,21].

The present study addresses this gap by systematically evaluating long-term degradation kinetics and microbiological quality of BP obtained via CM and UAM. EA of α-amylase, proteases, and lipases were monitored over 0–930 days at 10–40 °C, and statistical modeling was applied to assess method- and temperature-dependent effects. By integrating activity profiling, temperature-response analysis, and pharmacopoeial microbiological testing, this work provides a structured framework for understanding stability behavior in complex multienzyme preparations.

We hypothesized that UAM may influence long-term conformational stability and degradation kinetics of multienzyme systems.

## 2. Results

### 2.1. Effect of Storage Duration and Temperature on AA

#### 2.1.1. Storage Duration on AA (20 ± 1 °C; RH 35–40%)

**Figure 1:**
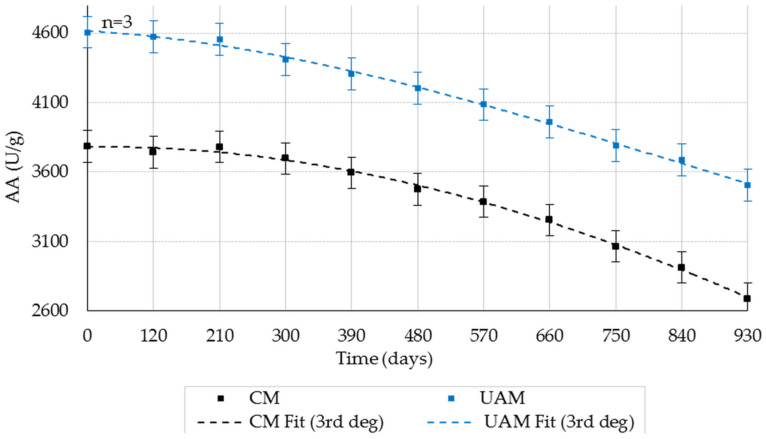
Effect of storage duration on AA (20 ± 1 °C; RH 35–40%) obtained by the CM and UAM. Mean values ± SD (*n* = 3). CM Fit and UAM Fit represent third-degree polynomial fits.

AA decreased over storage time in both CM and UAM preparations (Figure 1; mean ± SD, *n* = 3). For CM, the first pronounced decline occurred between 300 and 480 days (3698 → 3475 U/g), followed by additional marked reductions at 480–660 days and 660–750 days, with a final decrease to 2687 U/g at 930 days. For UAM, decreases were smoother: an early reduction was apparent at 210–300 days, with the most intensive decline between 300 and 480 days, followed by a more gradual decrease until 750 days and a final decline to 3505 U/g at 930 days. Overall, UAM retained higher AA than CM across the full 0–930-day period.

Two-way ANOVA (Table 1) showed significant effects of extraction method and storage duration on AA (*p* < 0.001 for both), as well as a significant method × duration interaction (F = 10.83, *p* = 5.66 × 10^−9^, partial η^2^ = 0.711), indicating different time-dependent degradation patterns between CM and UAM. The main effects were very large within the fitted model (method: F = 18,475.28, *p* = 2.22 × 10^−59^, partial η^2^ = 0.998; storage duration: F = 1688.39, *p* = 1.98 × 10^−53^, partial η^2^ = 0.997). Tukey’s post hoc test indicated significant AA reductions for CM starting at 120 days, whereas significant reductions for UAM became evident after 300 days (*p* < 0.05).

#### 2.1.2. Storage Duration × Temperature on AA

**Figure 2:**
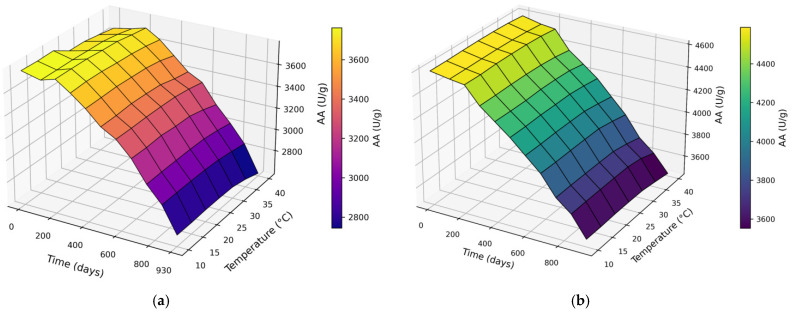
Effect of storage duration × Temperature on AA using the (**a**) CM and (**b**) UAM.

AA decreased with increasing storage duration at all tested temperatures (Figure 2a,b). For CM at day 0, AA ranged from 4587 to 4606 U/g, with slightly higher values at 20–30 °C and marginally lower AA at 40 °C. With increasing storage duration, AA declined progressively: at 120 days, AA decreased to 4547–4571 U/g (~0.6–1.1% loss vs. day 0); at 210–300 days, AA decreased to 4367–4557 U/g (~3–5% loss depending on temperature). During long-term storage (390–660 days), AA decreased further to 3900–4307 U/g. By 750–930 days, CM AA decreased to 3457–3791 U/g (overall loss ~17–25%), with the largest reductions consistently observed at 40 °C. Overall, higher storage temperatures (≥35 °C) accelerated AA loss, whereas moderate temperatures (approximately 20–25 °C) better preserved AA (Figure 2).

### 2.2. Effect of Storage Duration and Temperature on PA

#### 2.2.1. Storage Duration on PA (20 ± 1 °C; RH 35–40%)

**Figure 3:**
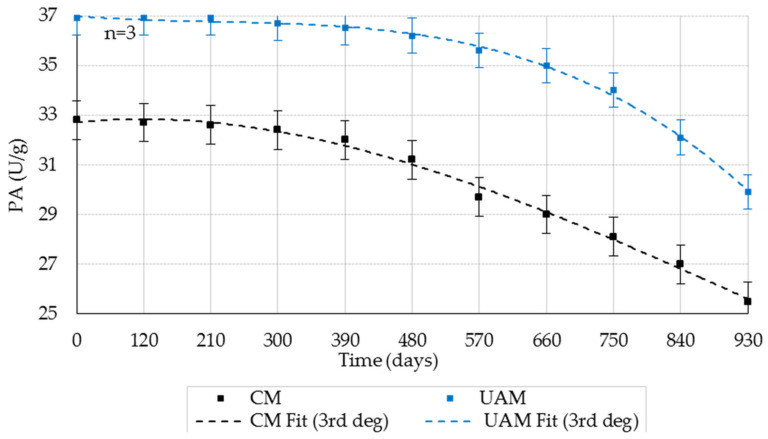
Effect of storage duration on the PA (20 ± 1 °C; RH 35–40%) obtained by the CM and UAM. Mean values ± SD (*n* = 3). CM Fit and UAM Fit represent third-degree polynomial fits.

PA decreased over time in both CM and UAM samples stored at 20 ± 1 °C, RH 35–40% (Figure 3; mean ± SD, *n* = 3). The decline occurred earlier and was more pronounced for CM than for UAM. Tukey’s HSD confirmed progressive time-dependent decreases, with earlier significant reductions for CM, whereas UAM retained significantly higher PA and exhibited delayed degradation during long-term storage (*p* < 0.05).

Two-way ANOVA (Table 2) indicated significant effects of method and storage duration and a significant interaction (*p* < 0.001 for all). Effect sizes (partial η^2^) were very large within the fitted model (method: 0.9980; storage duration: 0.9978; interaction: 0.9148), indicating strong separation between methods, a strong time effect, and method-dependent degradation kinetics.

#### 2.2.2. Storage Duration × Temperature on PA

**Figure 4:**
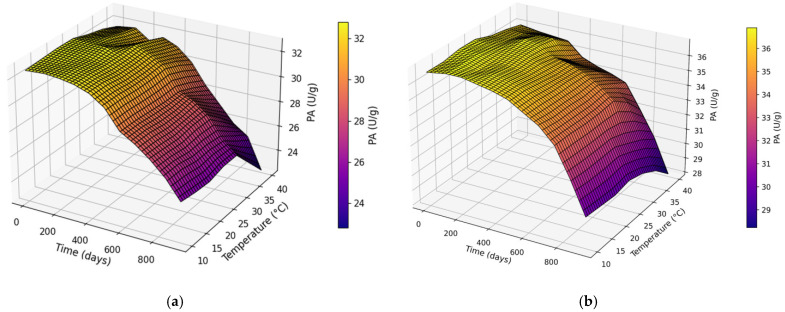
Effect of storage duration × temperature on PA using the (**a**) CM and (**b**) UAM.

PA declined with increasing storage duration for both CM and UAM across the tested temperatures (Figure 4a,b). Higher storage temperatures (35–40 °C) consistently resulted in lower PA values compared with lower temperatures, and the decline was more rapid for CM than for UAM. UAM maintained higher PA under identical time–temperature conditions and exhibited a less steep reduction during long-term storage, particularly at 10–25 °C.

### 2.3. Effect of Storage Duration and Temperature on LA

#### 2.3.1. Storage Duration on LA (20 ± 1 °C; RH 35–40%)

**Figure 5:**
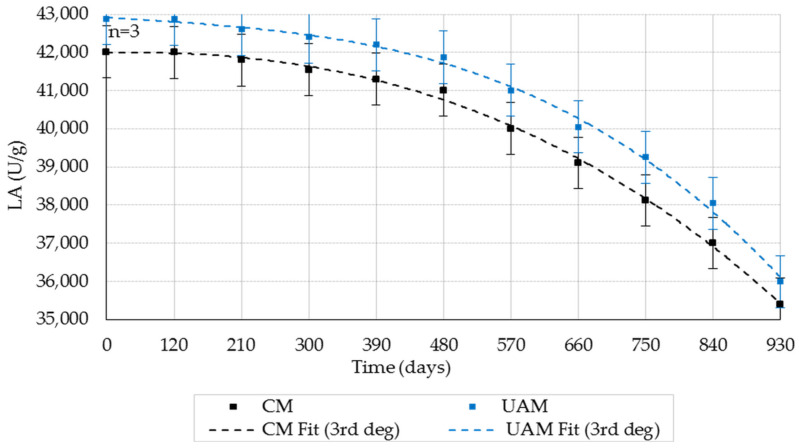
Effect of storage duration on the LA (20 ± 1 °C; RH 35–40%) obtained by the CM and UAM. Mean values ± SD (*n* = 3). CM Fit and UAM Fit represent third-degree polynomial fits.

LA decreased modestly during long-term storage for both CM and UAM (Figure 5; mean ± SD, *n* = 3). After 930 days, CM and UAM retained 84.3% and 83.9% of initial LA, respectively, corresponding to a total activity loss of ~15–16%.

Two-way ANOVA (Table 3) showed significant effects of storage duration and extraction method and a significant interaction (*p* < 0.001), indicating that LA degradation kinetics differed between CM and UAM. Storage duration showed the largest effect (F = 1957.22, partial η^2^ = 0.9978), and the method also contributed substantially (F = 859.01, partial η^2^ = 0.951), while the interaction term (F = 5.19, partial η^2^ = 0.541) indicated method-dependent time trends. Tukey post hoc analysis indicated that significant degradation became more evident after prolonged storage.

#### 2.3.2. Storage Duration × Temperature on LA

**Figure 6:**
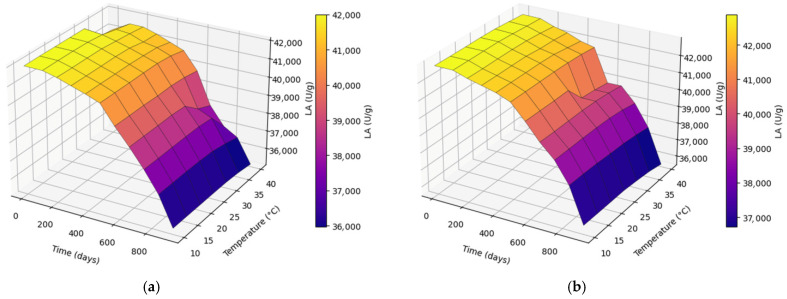
Effect of storage duration × temperature on LA using the (**a**) CM and (**b**) UAM.

LA decreased with increasing storage duration across all temperatures (Figure 6a,b). Temperature accelerated LA loss, with higher storage temperatures (35–40 °C) producing steeper declines than 10–25 °C. Both CM and UAM maintained comparatively higher LA at lower temperatures throughout storage, whereas the combination of prolonged storage and elevated temperature produced the lowest LA values. Across comparable storage conditions, the extraction method had a statistically significant effect on LA (*p* < 0.001), with UAM exhibiting slightly higher residual LA and a less pronounced time-dependent decline compared with CM, although the absolute difference between methods was modest.

##### 2.4. Microbiological Parameters of BP

Both CM and UAM complied with pharmacopoeial microbiological limits for non-sterile enzyme preparations [6,7]. Total aerobic microbial counts (TAMC) remained within the 10^3^ CFU/g range for both preparations. CM exhibited a mean TAMC of (1.55 ± 0.07) × 10^3^ CFU/g, while UAM showed (1.75 ± 0.06) × 10^3^ CFU/g (*p* = 0.017). Total yeast and mold counts (TYMC) were below 10^2^ CFU/g in both preparations, with CM showing 42.6 ± 1.7 CFU/g and UAM showing 11.7 ± 1.0 CFU/g (*p* < 0.001). All samples tested negative for *E. coli* and *Salmonella* spp.

The microbiological parameters of BP obtained by CM and UAM are presented in Table 4.

## 3. Discussion

### 3.1. Storage Duration and Temperature Effects on Enzymatic Stability

The 0–930-day dataset demonstrates a progressive decline in AA, PA, and LA in both CM and UAM preparations, confirming storage duration as the primary determinant of EA loss in BP. This time-dependent reduction is consistent with gradual conformational destabilization of protein components, including partial unfolding, disruption of intramolecular interactions, and accumulation of inactive structural states commonly reported for protein-based systems during long-term storage [1,2,3,4,5,22,23,24,25].

Two-way ANOVA revealed significant effects of extraction method, storage duration, and their interaction (*p* < 0.001), indicating method-dependent degradation kinetics. Elevated storage temperatures (35–40 °C) further accelerated activity decline compared with 10–25 °C, consistent with thermodynamic principles whereby increased molecular mobility lowers the effective energetic barrier between native and partially unfolded states, facilitating irreversible inactivation and aggregation processes [3,4,5,22,23,24]. The high partial η^2^ values reflect strong statistical separation within the controlled experimental design rather than absolute biological dominance of a single factor. However, no direct structural measurements were performed in this study.

### 3.2. Comparative Stability of CM and UAM Preparations

Across the storage period, UAM consistently retained higher residual AA and PA than CM, particularly at 20 ± 1 °C. After 930 days, AA was 3505 U/g in UAM versus 2687 U/g in CM, corresponding to substantially greater retained activity. These findings indicate kinetic stabilization in UAM-derived preparations, reflected by smoother degradation trajectories and lower apparent inactivation rates.

UAM enhances mass transfer and shortens processing time [17,18,19,20,21], potentially reducing exposure to destabilizing extraction conditions and limiting early structural stress. Although direct structural analyses were not performed, the functional data suggest that UAM may contribute to improved conformational resilience during storage. Verification of this hypothesis would require complementary techniques such as circular dichroism or calorimetric analysis.

### 3.3. Enzyme-Specific Stability Behavior

Distinct stability profiles were observed among the three EAs, reflecting the heterogeneous nature of BP. PA exhibited pronounced interaction effects, consistent with the known susceptibility of serine proteases to gradual inactivation and possible self-digestion during storage [11,12,13]. AA showed temperature sensitivity compatible with the structural role of Ca^2+^ in maintaining conformational rigidity.

In contrast, LA demonstrated comparatively high long-term stability, retaining approximately 84% of initial activity after 930 days in both preparations. Although the extraction method significantly influenced LA (*p* < 0.001), the magnitude of difference was moderate, indicating enzyme-specific stability hierarchies within the multienzyme system [25]. These findings emphasize that stability in complex enzyme preparations must be evaluated for each catalytic component rather than inferred from a single parameter.

### 3.4. Microbiological Quality

Both CM and UAM complied with pharmacopoeial microbiological limits for non-sterile enzyme preparations [6,7]. UAM exhibited significantly lower yeast and mold counts, while total aerobic counts remained within acceptable ranges for both methods. The absence of *E. coli* and *Salmonella* spp. confirms microbiological safety. Maintaining low microbial load is essential not only for regulatory compliance but also for preserving long-term functional stability.

### 3.5. Overall Implications

The integration of long-term kinetic monitoring, temperature-dependent analysis, and microbiological assessment provides a comprehensive evaluation of stability in BP. Storage at moderate temperatures (10–25 °C) favors preservation of EA. Within the limits of functional and statistical analysis, UAM demonstrated enhanced long-term residual activity compared with CM, suggesting method-dependent kinetic stabilization. Further studies incorporating direct structural characterization are required to elucidate the molecular mechanisms underlying these observations.

## 4. Materials and Methods

### 4.1. Materials and Chemicals

Fresh bovine pancreases (*n* = 41; total mass of 10.0 kg) were obtained from a certified meat supplier (S-Meat LLP, Almaty, Kazakhstan). Upon collection, the tissues were immediately stored in a portable automotive freezer HEERXIN DW-86W25 (HEERXIN, Shenzhen, China) at a controlled temperature of −5 to −7 °C to preserve enzymatic integrity prior to extraction. All analytical-grade reagents and chemicals used in this study, including buffers and substrates for enzymatic assays, were supplied by a local supplier (Veld LLP, Almaty, Kazakhstan).

### 4.2. Sample Preparation

Proteolytic EP were produced using the CM, which was based on a modified protocol described in patent [26] and later adapted in a separate peer-reviewed scientific study. Fresh bovine pancreas (10 kg), previously minced, was homogenized in 15 L of distilled water containing 75 mL of glacial acetic acid. The suspension was continuously stirred at 8–12 °C for 4 h to facilitate enzyme extraction, after which the liquid fraction was separated from residual tissue particles.

The remaining solid residue underwent a second extraction using an additional 5 L of the same acidic solution. After 30 min of agitation, the extract was recovered and combined with the primary extract. Calcium chloride (0.5 mol) was added to the pooled extracts, followed by supplementation with pancreatin at a concentration of 0.1 g/L. The pH was then adjusted to 8.1 using 20% NaOH, and EA was allowed to proceed for 24 h at 0–5 °C.

Subsequently, the pH of the mixture was lowered to 6.0 by adding 5 N hydrochloric acid. Insoluble components were removed by centrifugation using a separator. The enzymes were precipitated from the clarified supernatant using acetone, followed by drying under reduced pressure at 30–35 °C. The dried material was ground and sieved to obtain a homogeneous enzyme powder.

Although water activity was not measured directly, relative humidity during storage was maintained at 35–40% under controlled laboratory conditions. These parameters correspond to low moisture exposure and reduced risk of hydration-induced conformational mobility.

For the UAM, the protocol described above was modified by introducing an ultrasonic homogenization step to enhance tissue disruption and enzyme release. Ultrasonic treatment was performed using a Sonopuls HD 2200 processor (Bandelin electronic GmbH & Co. KG, Berlin, Germany) operating at 200 W in pulsed mode (1 s on/1 s off). The system functioned at a frequency of 20 kHz and was equipped with an MS 72 probe (13 mm tip diameter), which was immersed 1.5 cm into the sample.

Ultrasonic processing was applied for 15 min in total. To avoid thermal inactivation of enzymes, the temperature of the extraction medium was maintained below 25 °C using an external ice bath. The pulsed sonication regime was selected to reduce heat accumulation and preserve enzyme activity while maintaining effective cavitation. The pause intervals between pulses allowed dissipation of thermal energy and promoted bubble regeneration, thereby improving cavitation efficiency and cell disruption.

Although ultrasonic treatment can generate localized temperature increases, all extractions were carried out with the sample vessel placed in an ice-cooled water bath to limit thermal effects. Temperature was continuously monitored during sonication and remained below 25 °C. Additionally, EA assays were performed separately under strictly controlled thermal conditions using a thermostatic water bath. This ensured that any temperature-related changes in EA were associated exclusively with assay conditions rather than prior ultrasonic exposure.

Storage conditions were randomized to minimize potential positional or environmental bias. Samples from CM and UAM preparations were distributed randomly within temperature-controlled storage chambers, and their positions were periodically rotated at defined intervals. This approach ensured uniform exposure to storage conditions throughout the experimental period.

### 4.3. Thawing Loss

After thawing at 2 °C for 24 h, the samples were carefully dried with filter paper to remove residual surface moisture. The mass of each sample was measured prior to thawing (*m*1) and again after thawing (*m*2). The percentage of thawing loss was determined using the following equation:

(1) (m1−m2)/m1×100%

### 4.4. Determination of AA

All experimental measurements, data analyses, and interpretations of results were performed by certified specialists from the Kazakh Research Institute of Processing and Food Industry (KazRIPFI, Almaty, Kazakhstan), who possess substantial expertise in EA determination. The AA of pancreatin samples was evaluated according to the standard GOST 34440-2018, “Enzyme preparations for the food industry. Methods for determination of amylolytic activity” [27,28]. This analytical procedure is based on enzymatic hydrolysis of soluble starch into dextrins with different molecular weights under strictly controlled reaction conditions.

The hydrolysis process was carried out for 10 min using 6.0 units of enzyme activity at regulated pH and temperature parameters. One unit of AA was defined as the quantity of enzyme capable of converting 1 g of soluble starch into dextrins, corresponding to approximately 30–50% hydrolysis of the initial substrate. The resulting enzyme activity was expressed as units per gram of dry enzyme preparation (*AA*/*g*).

The degree of starch degradation was determined using a colorimetric assay based on iodine–starch complex formation. The reduction in iodine staining intensity reflected the decrease in the amount of residual, non-hydrolyzed starch. Absorbance measurements were performed using a Cary 60 UV-Vis spectrophotometer (Agilent Technologies, Santa Clara, CA, USA) with Cary WinUV software (version 5.2) operating within the wavelength range of 190–1100 nm.

The reagents used for activity determination included soluble starch, sodium acetate trihydrate, acetic acid, disodium phosphate, monopotassium phosphate, hydrochloric acid, and crystalline iodine. The thermostability of AA was further evaluated and expressed in enzyme units per gram according to the following equation:

(2) AA=6.6138×C−0.0192×d/n

where 6.6138 *and* 0.0192 are empirical coefficients obtained from a regression analysis of hydrolyzed starch mass versus enzyme mass per hour of enzymatic action; C—degree of starch hydrolysis; n—enzyme sample mass used in the analysis, g (adjusted for dilution); d—density of the EP (for liquid form), g/cm^3^.

All quantitative calculations were performed with an initial precision of one decimal place, followed by rounding to the nearest integer value, as the measured AA exceeded 100 U/g. Each reported value represents the mean of two independent determinations conducted under strictly controlled repeatability conditions.

Method performance was evaluated in accordance with established analytical validation criteria. The relative deviation between replicate measurements did not exceed ±7%, which confirms the high level of method precision and reproducibility. The applied analytical procedure ensured reliable quantification of AA and minimized potential systematic and random measurement errors.

### 4.5. Determination of PA

PA was determined according to GOST 34443-2018, “Enzyme Preparations for the Food Industry. Method for Determination of Proteolytic Activity” [29]. The method is based on enzymatic hydrolysis of bovine hemoglobin used as a natural protein substrate under controlled pH conditions, including acidic (pH 3.0), mildly acidic (pH 5.3), neutral (pH 7.0), and alkaline (pH 9.0) environments. During the reaction, the enzyme complex catalyzes the cleavage of hemoglobin molecules into low-molecular-weight peptides and free amino acids.

The enzymatic reaction was terminated by precipitation of the remaining non-hydrolyzed protein using trichloroacetic acid (TCA). The soluble peptides and amino acids formed during hydrolysis were subsequently quantified.

One unit of PA was defined as the amount of enzyme required to release peptides equivalent to 1 µmol of tyrosine per minute at 30 °C (1 µmol of tyrosine corresponds to 0.181 mg). The EA was expressed as units of PA per gram of dry sample (U/g).

The concentration of hydrolysis products was determined using a colorimetric assay based on the reaction between free amino groups and Folin’s reagent, resulting in formation of a blue chromophore. The absorbance of the reaction mixture was measured at 670 nm using a colorimetric analyzer (Shimadzu Corporation, Kyoto, Japan).

The analytical procedure employed lyophilized bovine hemoglobin as the substrate and tyrosine as the calibration standard. Additional reagents included Folin’s reagent, trichloroacetic acid, hydrochloric acid, orthophosphoric acid, glacial acetic acid, boric acid, urea, sodium carbonate, and sodium hydroxide.

PA values were calculated using the following equation:

(3) PA=D×4/TE×10×m×d

where D—optical density; 4—dilution factor after TCA addition; TE—tyrosine equivalent corresponding to the optical density of 1 µmol of tyrosine (determined from calibration curve); 10—hydrolysis time, min; m—mass of the EP used for analysis (based on a 1 cm^3^ working solution), g; d—density of the EP, g/cm^3^.

All calculations were performed with a precision of two decimal places and subsequently rounded to one decimal place, since the measured PA values were below 100 U/g. The reported activity values represent the arithmetic mean of two parallel determinations conducted under repeatability conditions.

### 4.6. Determination of LA

LA was determined in accordance with GOST 71139-2023 “Enzyme Preparations for the Food Industry. Method for Determination of Lipolytic Activity” [30]. The assay is based on enzymatic hydrolysis of triglycerides contained in high-oleic sunflower oil or olive oil, followed by quantification of released free fatty acids (FFA).

The substrate emulsion was prepared using high-oleic sunflower oil (tri-olein content ≥70%) stabilized with gum arabic. Initially, gum arabic solution was prepared by dissolving 33.0 g of gum arabic in 330 mL of distilled water under continuous stirring for 2 h, followed by centrifugation at 2000× *g* for 30 min to obtain a clarified solution.

The primary emulsion was prepared by mixing 36.5 g of oil with 330 mL of gum arabic solution and 30 mL of distilled water. The mixture was emulsified using a laboratory disperser (IKA Werke GmbH & Co. KG, Staufen, Germany) at 2000 rpm under cooling conditions (5–10 °C). Subsequently, homogenization was continued at 8000 rpm for 30 min while maintaining the temperature below 25 °C. The prepared emulsion was stored at 2–8 °C for no longer than two weeks.

The working emulsion was prepared immediately before analysis by mixing 20 mL of primary emulsion, 16 mL of buffer solution adjusted to the optimal pH of the tested lipase, and 23 mL of distilled water.

An enzyme solution equivalent to 625 U of LA was prepared by dissolving the appropriate amount of enzyme powder or liquid EP in buffer solution with optimal pH, followed by dilution to 25 mL. The enzyme solution was prepared freshly and used within the same day.

The working emulsion (59 mL) was pre-incubated at the optimal temperature for LA (35–50 °C depending on enzyme characteristics) for 15 min with continuous magnetic stirring. A control sample was obtained by transferring 5 mL of substrate into ethanol (96%) prior to enzyme addition.

The reaction was initiated by adding 1 mL of enzyme solution to the emulsion under constant stirring. Aliquots (5 mL) were withdrawn at defined time intervals (3, 5, 10, 15, 20, and 25 min) and immediately transferred into ethanol to terminate EA.

The amount of released free fatty acids was determined by potentiometric titration using 0.05 mol·dm^−3^ sodium hydroxide solution. Titration was performed using an automatic titrator equipped with a pH electrode. The equivalence point was determined from the maximum derivative of the titration curve.

The concentration of free fatty acids formed during hydrolysis was calculated using the following equation:

(4) CFFA = (Vs−Vb)×N×1000 / Ve

where Vs—volume of NaOH used for titration of the enzymatic sample (mL); Vb—volume of NaOH used for titration of the blank sample (mL); N—normality of NaOH solution; Ve—volume of emulsion sample (mL); 1000—conversion factor from dm^3^ to cm^3^.

LA was expressed as the amount of enzyme releasing 1 μmol of fatty acids per minute under the assay conditions.

The validity of the obtained results was verified by plotting the dependence of released free fatty acids concentration versus reaction time. The kinetic curve was expected to demonstrate a typical logarithmic increase followed by a plateau, confirming correct enzyme concentration and assay conditions. If deviations from the expected kinetic profile were observed, the assay was repeated with adjusted enzyme concentration.

Measurements were performed using an automatic titrator equipped with dynamic titration mode and pH electrode calibrated with certified buffer solutions (±0.03 pH units). All analyses were conducted at ambient laboratory conditions (20 ± 5 °C; RH 35–40% (controlled). Magnetic stirrers with thermostatic water bath ensured temperature stability within ±1 °C during reactions.

The uncertainty of titration was evaluated based on repeatability of NaOH volume measurements and electrode calibration stability. The relative standard deviation of replicate titrations did not exceed ±2.5%, and the expanded measurement uncertainty (k = 2) was estimated at ±3.8% of the reported LA value. The detection limit of the titration method, calculated based on blank variability and minimal detectable NaOH volume difference, corresponded to 0.5 µmol of released fatty acids under the applied conditions.

### 4.7. Determination of MP

MP of the tested samples was evaluated in accordance with the General Pharmacopoeial Monograph of the State Pharmacopoeia of the Russian Federation, XIV Edition, 1.2.4.0002.18 [6,7], harmonized with international pharmacopoeial standards for non-sterile medicinal products.

TAMC and TYMC were determined using the plate count method. Sample aliquots were aseptically diluted in sterile buffered solution to obtain appropriate dilutions for quantitative analysis. Each dilution was inoculated onto suitable nutrient media [6,7].

TAMC was determined using casein–soybean digest agar, followed by incubation at 30–35 °C for 3–5 days. TYMC was evaluated using Sabouraud dextrose agar, with incubation at 20–25 °C for 5–7 days. After incubation, visible colonies were counted and expressed as colony-forming units per gram or milliliter of sample (CFU/g or CFU/mL) [6,7].

Qualitative detection of specified microorganisms potentially hazardous to human health was performed using selective enrichment and differential media. The analysis included screening for indicator bacteria typically regulated for non-sterile pharmaceutical products. Enrichment broths and selective agar media were used according to pharmacopoeial requirements, followed by incubation under recommended temperature and time conditions. Suspected colonies were confirmed by morphological evaluation and standard biochemical identification procedures.

All microbiological procedures were performed under aseptic conditions. Growth promotion testing of culture media and suitability testing of the method were conducted to confirm the reliability of microbial recovery. Negative and positive controls were included in each analytical series to ensure method accuracy and reproducibility.

Microbial limits were evaluated in accordance with pharmacopoeial acceptance criteria for non-sterile medicinal products.

The limit of detection for TAMC and TYMC corresponded to 10 CFU/g, based on the lowest dilution plated and pharmacopoeial methodology. For qualitative detection of specified microorganisms (*E. coli and Salmonella* spp.), the detection limit corresponded to the absence of growth in 1 g of sample under enrichment conditions, in accordance with pharmacopoeial requirements.

### 4.8. Statistical Analysis

All experiments were performed in triplicate, and the results are expressed as mean ± SD. Statistical significance of main effects (*p* < 0.05) was assessed using two-way ANOVA. Normality of residuals was verified using the Shapiro–Wilk test, and homogeneity of variances was evaluated using Levene’s test. Post hoc multiple comparisons were conducted using Tukey’s HSD test with adjusted *p*-values. Effect sizes (partial η^2^) were calculated to estimate the magnitude of observed effects within the controlled experimental design.

To describe the relationship between EA and storage duration or temperature, third-order polynomial regression models were applied for graphical trend analysis. The general form of the model was

(5) Y=aT3=bT3+cT+d

where Y—represents EA (U/g); T—denotes temperature (°C) or storage duration (days); and a, b, c, and d are regression coefficients determined using the least squares method.

These polynomial models were used exclusively for descriptive curve fitting and visualization of degradation trajectories and were not applied for hypothesis testing.

## 5. Conclusions

This study systematically evaluated the long-term stability (0–930 days) of BP obtained by CM and UAM, integrating enzymatic kinetics, temperature–time modeling, and microbiological assessment. Storage duration was identified as the dominant factor governing AA, PA, and LA decline, while elevated temperatures (35–40 °C) significantly accelerated inactivation. Two-way ANOVA confirmed significant effects of extraction method, storage duration, and their interaction, indicating method-dependent degradation kinetics. UAM preparations retained higher residual AA and PA and exhibited smoother degradation trajectories, consistent with improved kinetic stability; however, direct structural validation was not performed. LA demonstrated comparatively high long-term resilience (~84% retention after 930 days). Both preparations complied with pharmacopoeial microbiological criteria, with UAM showing significantly reduced fungal counts and improved fungal control while maintaining acceptable total aerobic counts. These findings suggest kinetic stabilization possibly associated with enhanced conformational resilience of enzyme components during prolonged storage, although direct structural characterization will be required to confirm the underlying molecular mechanisms. Overall, UAM represents a technologically advantageous strategy for enhancing the long-term stability and microbiological robustness of BP.

While the present study provides comprehensive insights into the long-term stability of BP under defined storage conditions, certain limitations should be acknowledged. Continuous monitoring of water activity and potential structural changes in enzymes were not performed, and the findings are based primarily on activity-based assays. Additionally, storage conditions were controlled in the laboratory and may not capture all variations encountered in industrial or real-world settings. Future work incorporating broader physicochemical analyses and more diverse storage scenarios could further elucidate the mechanisms affecting enzyme stability.

## 6. Future Perspectives

The principal limitation is the absence of direct structural measurements. Future investigations combining spectroscopic (CD), calorimetric (DSC), and aggregation-sensitive (SEC, DLS) techniques are necessary to quantify changes in secondary structure content, melting transitions, and oligomeric state distributions. Such data would enable direct correlation between conformational stability landscapes and the kinetic behavior observed during prolonged storage.

## Figures and Tables

**Table 1:** Two-way ANOVA for AA (CM vs. UAM) as a function of storage duration.

Source	df	SS (U^2^/g^2^)	MS (U^2^/g^2^)	F	*p*-Value	Partial η^2^
Method	1	9.51 × 10^6^	9.51 × 10^6^	1.84 × 10^4^	2.22 × 10^−59^	0.998
Storage duration	10	8.69 × 10^6^	8.69 × 10^5^	1.69 × 10^3^	1.98 × 10^−53^	0.997
Method × Storage duration	10	5.57 × 10^4^	5.57 × 10^3^	1.08 × 10^1^	5.66 × 10^−9^	0.711
Error	44	2.27 × 10^4^	5.16 × 10^2^	-	-	-

**Table 2:** Two-way ANOVA for PA (CM vs. UAM) as a function of storage duration.

Source	df	SS (U^2^/g^2^)	MS (U^2^/g^2^)	F	*p*-Value	Partial η^2^
Method	1	3.87 × 10^5^	3.87 × 10^5^	2.18 × 10^4^	5.72 × 10^−61^	0.9980
Storage duration	10	3.51 × 10^2^	3.51 × 10^1^	1.98 × 10^3^	5.90 × 10^−55^	0.9978
Method × Storage duration	10	8.38 × 10^0^	8.38 × 10^−1^	4.73 × 10^1^	3.12 ×10^−20^	0.9148
Error	44	7.80 × 10^−1^	1.77 × 10^−2^	-	-	-

**Table 3:** Two-way ANOVA for LA (CM vs. UAM) as a function of storage duration.

Source	df	SS	MS	F	*p*-Value	Partial η^2^
Method	1	1.32 × 10^7^	1.32 × 10^7^	8.59 × 10^2^	<1.00 × 10^−4^	0.951
Storage duration	10	3.01 × 10^8^	3.01 × 10^7^	1.96 × 10^3^	<1.00 × 10^−4^	0.9978
Method × Storage duration	10	7.97 × 10^5^	7.97 × 10^4^	5.19 × 10^0^	<1.00 × 10^−3^	0.541
Error	44	6.76 × 10^5^	1.54 × 10^4^	-	-	-

**Table 4:** Microbiological Parameters of BP (CM and UAM).

Parameter	CM (Mean ± SD)	UAM (Mean ± SD)	*p*-Value	Hedges’g
TAMC (CFU/g)	(1.55 ± 0.07) × 10^3^	(1.75 ±0.06) × 10^3^	0.017	2.55
TYMC (CFU/g)	42.6 ± 1.7	11.7 ± 1.0	<0.001	17.89
*E. coli*	ND	ND	-	-
*Salmonella* spp.	ND	ND	-	-

Notes: Values are mean ± SD of three independent determinations (*n* = 3). Two-tailed Student’s t-test; effect size by Hedges’ g. ND, not detected (below detection limit).

## Data Availability

The data presented in this study are available on request from the corresponding author.
